# Reduction in sugar drink valuation and consumption with gamified executive control training

**DOI:** 10.1038/s41598-023-36859-x

**Published:** 2023-06-30

**Authors:** Hugo Najberg, Michael Mouthon, Géraldine Coppin, Lucas Spierer

**Affiliations:** 1 Laboratory for Neurorehabilitation Science, Medicine Section, Faculty of Science and Medicine, University of Fribourg, 1700 Fribourg, Switzerland; 2 UniDistance Suisse, Schinerstrasse 18, 3900 Brigue, Switzerland; 3 Swiss Center for Affective Sciences, University of Geneva, 1205 Genève, Switzerland

**Keywords:** Neuroscience, Cognitive control

## Abstract

**Abstract:**

The overvaluation of high-energy, palatable food cues contributes to unhealthy eating and being overweight. Reducing the valuation of unhealthy food may thus constitute a powerful lever to improve eating habits and conditions characterized by unhealthy eating. We conducted a double-blind, placebo-controlled, randomized intervention trial assessing the efficacy of a five to twenty days online cognitive training intervention to reduce sugary drink perceived palatability and consumption. Our intervention involved a recently identified action-to-valuation mechanism of action, in which the repeated inhibition of prepotent motor responses to hedonic food cues in a Go/NoGo (GNG) and an attentional bias modification (ABM) task eventually reduces their valuation and intake. Confirming our hypotheses, the experimental intervention with consistent (100%) mapping between motor inhibition and the targeted unhealthy sugary drinks cues induced a larger decrease in their valuation than the control intervention with inconsistent (50%) mapping (− 27.6% vs. − 19%), and a larger increase of the (water) items associated with response execution (+ 11% vs + 4.2%). Exploratory analyses suggest that the effect of training on unhealthy items valuation may persist for at least one month. Against our hypothesis, we observed equivalent reductions in self-reported consumption of sugary drinks following the two interventions (exp: − 27% vs. ctrl: − 19%, BF_01_ = 4.7), suggesting a dose-independent effect of motor inhibition on self-reported consumption. Our collective results corroborate the robustness and large size of the devaluation effects induced by response inhibition on palatable items, but challenge the assumption of a linear relationship between such effects and the actual consumption of the target items.

**Protocol registration:**

The stage 1 protocol for this Registered Report was accepted in principle on 30/03/2021. The protocol, as accepted by the journal, can be found at: 10.17605/OSF.IO/5ESMP.

## Introduction

The overvaluation of high-energy density palatable food cues represents a key contributor to unhealthy eating and fosters the development and maintenance of many prevalent diseases^1^, ranging from obesity^2^ to diabetes^3^ and cancer^60^. Food cue overvaluation, for instance, predicts and drives food overconsumption in individuals with excessive weight^4–8^.

Restoring healthy neurocognitive responses to food cues would thus constitute a potent lever to reduce excessive weight and related diseases. Recent literature suggests that this aim could be achieved via an ‘action-to-valuation’ mechanism of action; indeed, the repeated execution and inhibition of motor responses to food items modulate their perceived value and consumption (for systematic reviews, see Refs.^9–12^).

In the present study, we tested the efficacy of an intervention involving this mechanism of action with a double-blind, placebo-controlled, non-crossover, randomized intervention trial in which we assessed whether a recommended one-month parallel practice of Go/NoGo (GNG) and an attentional bias modification (ABM) task decreased soda items’ valuation (as indexed by their palatability) and real-world consumption.

In the GNG, we instructed the participants to respond as quickly as possible to a set of stimuli (the Go stimuli) while withholding their responses to another set of stimuli (the NoGo). With practice, NoGo stimuli end up automatically triggering motor inhibition processes. In turn, automatic approach responses and/or elevated motor excitability toward reward-associated stimuli may be reduced^11^. In addition, feedback loops from motor (inhibition) to reward areas, and/or the automatic engagement of the avoidance/aversive center developing with the learning of the association between the NoGo stimuli and motoric inhibition, may also reduce reward responses, and in turn stimuli valuation^13–17^.

For the ABM task, the participants have to respond as fast as possible to items when a Go-cue is displayed and before it disappears^18^. Since paying attention to the items associated with the cues (but not to those not associated with the cue) improves task performance, attention is eventually automatically allocated to (or withheld from) the cued items^19^. The development of attentional bias increases or decreases the saliency and perceived value of the target items^20–22^, as well as their consumption^23,24^.

Based on this literature, we posit that the associative learning of inhibition to NoGo soda cues, and the development of attentional bias away from soda cues, would synergistically reduce their value and consumption. An opposite effect might manifest for the other category of non-soda cues (low-sugar water or flavored water), which would promote the replacement of soda by healthy beverages and not merely reduce soda consumption, thereby ensuring the maintenance of hydration^25^.

We focused on high sugar-density drinks (i.e., soda, iced tea, energy drinks) because as a source of sugar, these beverages represent a major contributor to people being overweight^26^, affecting 40% of adults and 20% of children in Switzerland, plus 400,000 individuals suffering from diabetes (Federal Statistical Office data). Each Swiss citizen drinks, on average, 82 L of soda/energy drinks, one of the highest consumptions in Europe. A comparable problem is observable in most Western countries.

Our design extends and improves previous attempts at testing the efficacy of value mechanisms at several levels. First, most prior research has only involved a short, single laboratory training session, unlikely to reveal the full efficacy of this type of intervention on eating behavior^27–32^. For our intervention, we instructed the participants to engage in each task for 10 min per day for a recommended four weeks. Second, task gamification should improve participants’ motivation and their adherence to the intervention; we did not only present the cognitive training tasks in artistic auditory and visual environments, but also introduced reward mechanisms (an internal economy based on performance, comparisons with peers, etc.). In addition, we have set up a system of progressive difficulty to keep the training tasks challenging, as well as adjusted to participants’ performance and improvement with practice. Finally, we have created a system to individualize the intervention stimuli to each participant’s tastes and drinking habits by targeting their preferred high sugar-density drink items, as measured with palatability scales at the beginning of the intervention.

Importantly, we managed to solve the pervasive problem of expectations in cognitive interventions by using a so-called ‘mechanistic’ control group*:* we compared the effects of the experimental intervention with those measured in a control group, who will take part in the exact same training program, except that the probability of the association between the unhealthy drink cues and the inhibition of motor responses (i.e., the ‘active ingredient’ of the experimental intervention) is neutralised. While this type of control group is not new^30,33^, it has been underutilized. In the control group, both the Go and NoGo signals had the same probability of cuing an unhealthy or a healthy item (see the “Materials and methods” section).

Hence, since both groups practiced the same task, they both expected to improve in the same cognitive processes. We do not expect the participants to detect the difference in the stimulus–response contingencies, as each participant only practiced one version of each task. Regardless of how a participant would interpret the stimulus–response contingency, this should not confound the outcome, because we explicitly informed both groups that we expected the intervention to reduce the perceived value of the unhealthy drink cues, and reversely for the healthy drink cues. In this way, even if more participants in the experimental group (versus the control group) detect the contingencies and form related expectations, the expectations in the control group were also set high and in the same direction.

We first posited a larger decrease in unhealthy items’ palatability ratings (compared to the healthy items) in the experimental group than in the control group between the pre- and post-intervention periods (Hypothesis 1; H1). We tested this hypothesis with the triple interaction term of the between-subjects factor Intervention (Experimental; Control), and the within-subject factors Session (Pre-intervention; Post- intervention), and Drink Type (Heathy; Unhealthy). Two Intervention by Session interaction planned contrasts further tested for a larger decrease in unhealthy drink palatability (H1a) and an increase in healthy drink palatability (H1b).

Next, we posited that the participants’ reported drinking consumption would show a larger reduction in the experimental group than in the control group after the intervention (Hypothesis 2; H2). We tested this hypothesis with the interaction of the factor Intervention (Experimental; Control) and Session (Pre-intervention; Post-intervention). We chose a pre- vs. post-intervention design for H2 instead of a regression model as this design allows us to detect any effect independently of which shape the curve takes during training.

Finally, we expected the change in unhealthy item ratings to mediate the change in consumption (Hypothesis 3; H3). See Table 1 for a summary of the hypotheses and their respective analysis plans.

**Table 1:** Design table.

Question	Hypothesis	Sampling plan to reach 0.95 power (power analysis)* and minimum effect size of interest	Analysis plan	Interpretation given to different outcomes
Will the intervention decrease unhealthy items’ ratings while improving healthy items’ palatability ratings?	H1: A larger decrease in unhealthy items’ palatability ratings (as compared to the healthy items) in the experimental than control group between the pre- and post- intervention	102 participants (based on Monte Carlo simulations; see the ‘Sampling plan’ section) after data exclusion Minimum effect size of interest is of 5/100	If the homoscedasticity assumption is respected, then two-sided parametric mixed analysis of variance (ANOVA) with the between-subjects factor Intervention (experimental; control) the within-subjects factor Session (pre-intervention; post-intervention), and Drink Type (heathy; unhealthy). If the homoscedasticity assumption is violated, then non-parametric ANOVA will be performed on the same factors	If p < 0.05 for the triple interaction term, then the intervention has a different effect than the control group, which is different for one of the drink types. In the case that p > 0.05, BF_01_ should be above 3 to support the absence of interaction
Will the intervention decrease unhealthy items’ palatability ratings?	H1a: A larger decrease in unhealthy items’ palatability ratings in the experimental group (versus the control group) between the pre- and post- intervention	212 participants (based on Monte Carlo simulations independent of H1; see the ‘Sampling plan’ section) after data exclusion Minimum effect size of interest is of 5/100	Same IF–THEN rule as H1 Two-sided mixed ANOVA with the between-subjects factor Intervention (experimental; control) and the within-subjects factor Session (pre-intervention; post- intervention)	Same equivalence rule as H1 If p < 0.05 for the interaction term, then the intervention has a different effect on the experimental (versus the control group) regarding the change in unhealthy items’ palatability ratings
Will the intervention increase healthy items’ palatability ratings?	H1b: A larger increase in healthy items’ palatability ratings in the experimental (versus the control group) between the pre- and post- intervention	212 participants (based on the same Monte Carlo simulations as H1a; see the ‘Sampling plan’ section) after data exclusion Minimum effect size of interest is of 5/100	Same IF–THEN rule as H1 Two-sided mixed ANOVA with the between-subjects factor Intervention (experimental; control) and the within-subjects factor Session (pre-intervention; post- intervention)	Same equivalence rule as H1 If p < 0.05 for the interaction term, then the intervention has a different effect on the experimental (versus the control group) regarding the change in healthy items’ palatability ratings
Will the intervention reduce the reported consumption of high sugary drinks?	H2: A larger reduction in consumption of high sugary drinks in the experimental group (versus the control group) between the pre- and post- intervention	210 participants (analytical power analysis; Cohen’s method; see the ‘Sampling plan’ section) after data exclusion Minimum effect size of interest is a partial Cohen’s f of 0.25	Same IF–THEN rule as H1 Two-sided mixed ANOVA with the between-subjects factor intervention (experimental; control) and the within-subjects factor session (pre-intervention; post- intervention)	Same equivalence rule as H1 If p < 0.05 for the interaction term, then the intervention has a different effect on the experimental (versus the control group) regarding the change in high sugary drink consumption
Does the change in unhealthy items’ palatability ratings mediate the change in the consumption of unhealthy drinks?	H3: The training leads to a change in unhealthy items’ palatability ratings, which in turn leads to a change in their reported consumption	104 participants (analytical power analysis; Vittinghoff, Sen and McCulloch’s method; see the ‘Sampling plan’ section) after data exclusion Both H1a and H2 must reach their minimum effect size of interest	If H1a and H2 reach significance, then use mediation analysis with 1,000 bootstrapping to estimate the causal mediation effect of the mediator (the change in unhealthy items’ palatability ratings) for the outcome model (the change in unhealthy drinks’ consumption)	If p < 0.05 for the average causal mediation effect, then the change in unhealthy drinks’ palatability ratings causes the change in their reported consumption

*The sample sizes mentioned for each hypothesis are the minimum sample size needed to reach a sufficient power for their respective analysis, and may be smaller or equal to the final sample size. The sample size in Table 1 were mistakenly not updated in the Stage 1 manuscript after the planned power was changed from 90 to 95%.

## Materials and methods

### Ethics information

All procedures were approved by the *Commission cantonale (VD) d’éthique de la recherche sur l’être humain* (CER-VD; protocol #2021-00884), were performed in accordance with the relevant guidelines and regulations, and in accordance with the Declaration of Helsinki. We compensated the participants for their participation with a lottery system, attributing prices depending on their final position on the group-level score distribution. The procedure was conducted fully online. All participants signed an informed consent form before any data were collected.

### Design

#### Recruitment and screening

The participants were recruited through a public advertisement.

The inclusion criteria included:

- 18-to-45-year-old healthy individuals
- Regular soda drinker (at least 3 glasses of 20 cl a week), as assessed by the soda drinking frequency item of our custom health questionnaire.

Participants were excluded if they:

- Had a past or current diagnosis of eating disorders
- Planned to have an active restrictive dieting in the next 4 months
- Previously participated in a food-related executive control training study
- Lost or gained of at least 10% of their weight in the last 6 months
- Had any olfactory or gustative impairments (including smokers of more than 10 cigarettes per day).

#### General procedure

Part of the method of the present study is identical to a registered report from our group^34^. Differences between the two protocols are in the absence of face-to-face screening in a laboratory, the inclusion of consumption frequency questionnaires, and the nature of the trained stimuli. For the sake of clarity, we did not systematically rephrase the methods descriptions shared between the two studies.

The study was carried out fully online. The recruitment procedure directed the participants to an online website to sign informed consent forms. Once done, the website was screened for inclusion/exclusion criteria using a custom health and drinking frequency questionnaire. They then were randomly assigned to one of the control or experimental interventions. We provided them a link to download the assigned intervention, along with a booklet explaining it. The exact hypotheses of the intervention were explained to every participant in both groups (i.e., reducing the valuation of unhealthy items and in turn reducing their consumption). However, we did not inform them of the existence of the control group or the rationale behind the stimulus–response mapping rules.

Our laboratory engineer randomly renamed the experimental and control interventions Condition 1 and Condition 2, thus preventing the experimenter from inferring the participants’ condition assignment, and in turn ensuring double blinding during the data analyses.

Before they could start the training, all participants had to report their soda consumption from the past day and completed the analogue scales of the items’ valuation on their device. We only used the highest rated healthy and unhealthy items (above the median) during the training.

We asked the participants to report their soda consumption from the past day on two random weekdays for each week of the intervention.

The participants were asked to train for a minimum of 20 min (a minimum of 10 min of GNG and a minimum of 10 min of ABM), 5 days a week for 4 weeks, in order to complete the intervention. At any time after the fifth completed day of training, the participants were able to end the intervention, fill out the same analogue scales of item palatability, and then be redirected on the website to fill out the core module of the Game Experience Questionnaire^35^ and a custom-made debriefing questionnaire. They were not able to end the intervention before completing five days of training, and were forced to end the intervention after twenty days of training. The procedure first required the participants to complete a full month of training, but pilot data revealed this requirement to be too challenging. Hence, since recent literature indicates that shorter interventions induce corresponding effect size (e.g. Ref.^10^), the initial instruction was eventually replaced by a recommended month of training, with a minimum of five days (the editor approved the change in training length before the beginning of data collection).

#### Item valuation

Within the intervention software, the participants rated 50 randomly ordered pictures of high sugar-density drinks using visual analogue scales with the question ‘Imagine drinking this; how much do you appreciate it?’. The scale ranges from ‘not at all’ to ‘very much’ (0 and 100 points, respectively), with a marker in the middle (50 points). A blue arrow indicated where the participants have responded. Post-intervention, only the trained items (i.ethose above the median at pre-intervention) were displayed again.

The participants were instructed to rate each item intuitively^36^ after waking up and before eating to improve the reliability of the measures.

#### Consumption frequency

**Figure 1:**
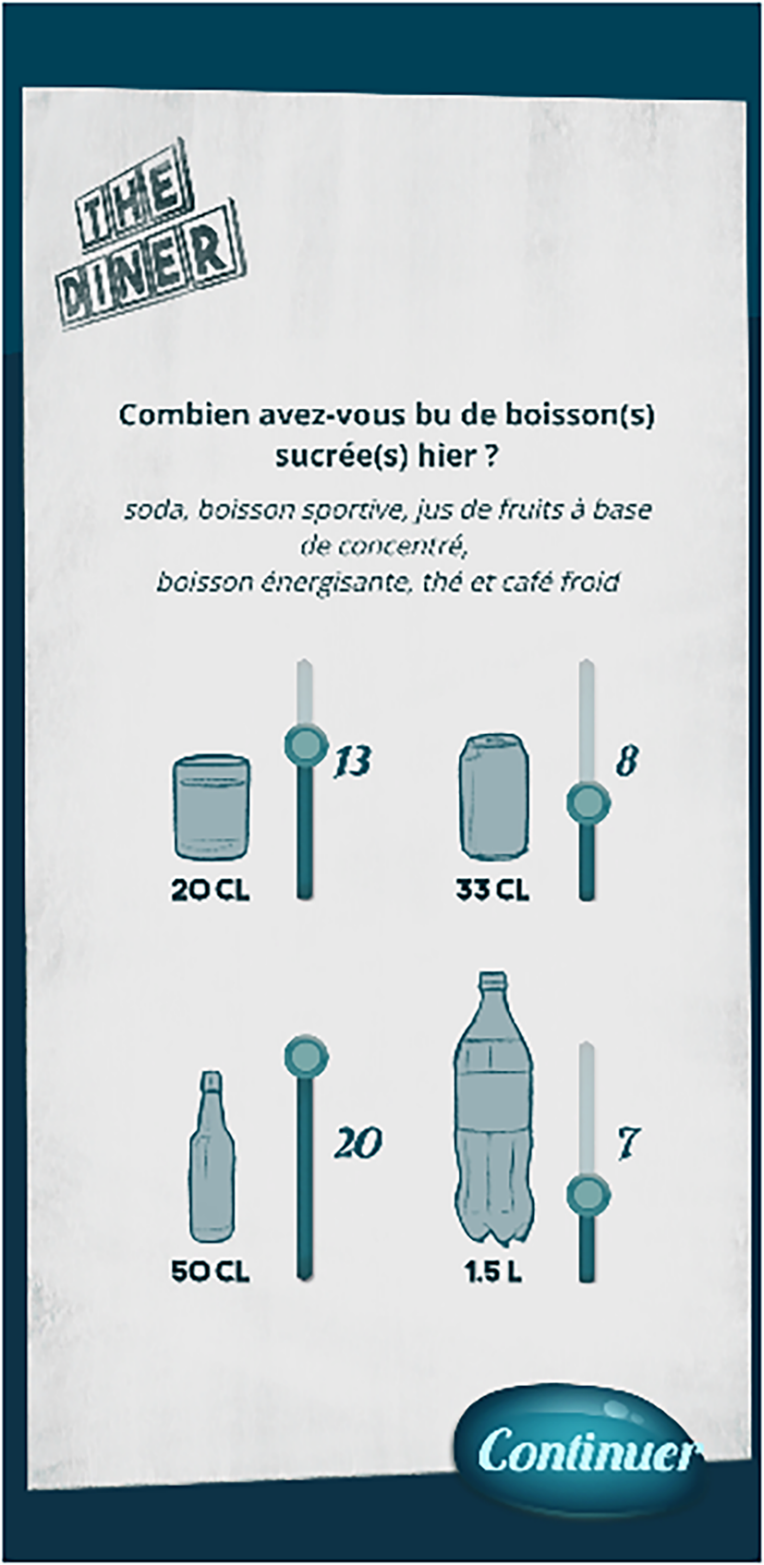
Screenshot of the consumption frequency questionnaire. The following questions were asked: “How many sugary beverages did you drink yesterday”/“soda, sport drink, juice made from concentrate, energy drink, ice tea, and iced coffee”.

Twice a week, randomly from Tuesday to Saturday, the participants were asked after the first block to report on their soda consumption from the previous day within the intervention software. If a participant did not play the randomly chosen day, the consumption report was asked the following day. They answered the question ‘How much soda did you drink yesterday?’ by indicating how many glasses, cans, small and/or large bottles they drank with the help of pictures (Fig. 1). The measure was then translated into litres (glass = 0.2 L, can = 0.33 L; small bottle = 0.5 L, large bottle = 1.5 L). We used the first and last measures for the pre- versus post-intervention analysis (H2; see ‘Analysis plan’), but we still recorded the in-between measures as additional exploratory data.

#### Stimuli

We focused on drinks because these items show marked individual preferences, their consumption can be reliably measured due to reduced variability in packaging size, and the limited number of brands enables the generation of stimuli matching real-world presentations.

The stimuli were drinks marketed in Switzerland: 43 pictures of unhealthy drinks, 7 pictures of water bottles, and 6 pictures of aromatised water bottles taken by a professional photographer. The pictures can be downloaded on the OSF study page (10.17605/OSF.IO/5ESMP).

#### Training tasks

The gamified intervention was implemented as an Android and iOS application developed on the 2019 version of Unity software.

The participants were able to train for the GNG and ABM tasks at their own pace as long as the 10 min mark is reached for each task. In both tasks, the participants had to complete as many trials they can in one session. With each correct response, the task difficulty increased. After making a certain number of accuracy or speed errors, the run was over.

Table 2 summarises the task parameters. Table 3 depicts the percentages of healthy, unhealthy, and neutral items based on the trial condition and task.

**Table 2:** Task-specific parameters.

	GNG	ABM
Go/NoGo rate	70% Go 30% NoGo	25% Go (cued items) 75% NoGo (non-cued items)
Stimulus duration	1.25 s maximum and disappearing after the response	
Feedback duration	250 ms	
Visual cue duration	Until item offset	
Visual cue delay	50 ms	Go Signal Delay (GSD): based on difficulty level (see Table 4)
Auditory cue duration	300 ms	NA
Auditory cue delay	100 ms	NA
Interstimulus interval (ISI)	1000–2000 ms	800–1300 ms*

*Since the participants only respond to 25% of the trials during the ABM, we reduced its ISI to prevent boredom.

**Table 3:** Proportion of item categories displayed for each trial condition and group.

Experimental group			
Trial condition	Item type		
	Healthy	Unhealthy	Neutral
Go trials	80%	0%	20%
NoGo trials	0%	80%	20%

Control group			
Go trials	40%	40%	20%
NoGo trials	40%	40%	20%

**Table 4:** Difficulty parameters at each level for all tasks (in seconds).

	1	2	3	4	5	6	7	8	9	10	11	12	13	14	15	16	17	18
GNG (RTT)	1.1	1	0.9	0.8	0.725	0.675	0.625	0.575	0.55	0.525	0.5	0.475	0.452	0.43	0.407	0.387	0.36	0.33
ABM (1.25-GSD)	0.88	0.81	0.74	0.67	0.62	0.57	0.53	0.49	0.455	0.42	0.39	0.36	0.335	0.31	0.29	0.27	0.26	0.25

To minimise the potential effect of the larger associative uncertainty in the control group (versus in the experimental group) on motivation and responding^37–40^, as well as participants’ expectations for the aim of the study, we added neutral distractor drink items to both the experimental and control groups.

##### Go/NoGo

**Figure 2:**
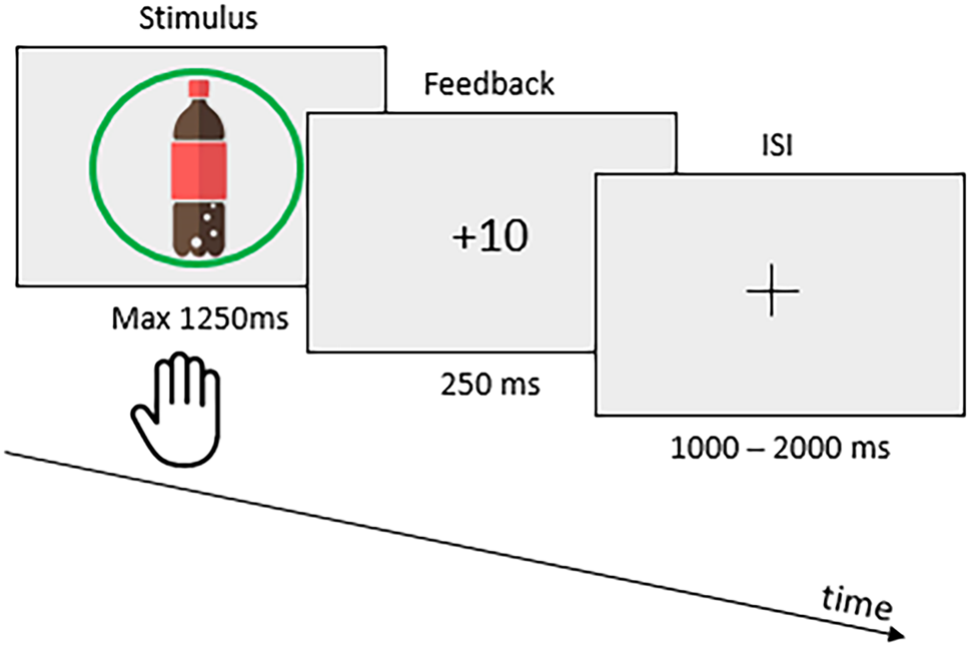
Schematic Go/NoGo task timeline, depicting the sequence of stimuli and response window.

For the GNG task, the participants were presented with drink pictures and instructed to drag the pictures that are circled in green and accompanied by a high-pitched tone (1,000 Hz, 300 ms duration, 100 ms post-stimuli onset) as fast as possible to the bottom of the screen; they must not touch the pictures circled in red that are accompanied by a low-pitch tone (400 Hz^28^). A correct response was defined either by responding to green-cued pictures (hit) below the reaction time threshold (RTT) or not responding to red-cued pictures (correct rejection [CR]). In these situations, positive green feedback (i.e., the points obtained) was displayed with a rewarding sound. In the case of a hit above the RTT, negative orange (‘too late’) feedback was displayed. If they responded to a red-cued picture (false alarm [FA]) or withhold response to a green-cued picture (miss), a negative red cross was displayed as feedback (Fig. 2). The Go and NoGo cues were delayed by 50 ms after stimulus onset for the picture to be treated by the participants’ visual system before they saw the item’s condition. This delay prevented the participants from only treating the cue without giving attention to the item.

To ensure response potency (i.e., a high pre-activation of motoric response), 70% of the trials consisted of Go items, and 30% of NoGo items.

##### Attentional bias modification

**Figure 3:**
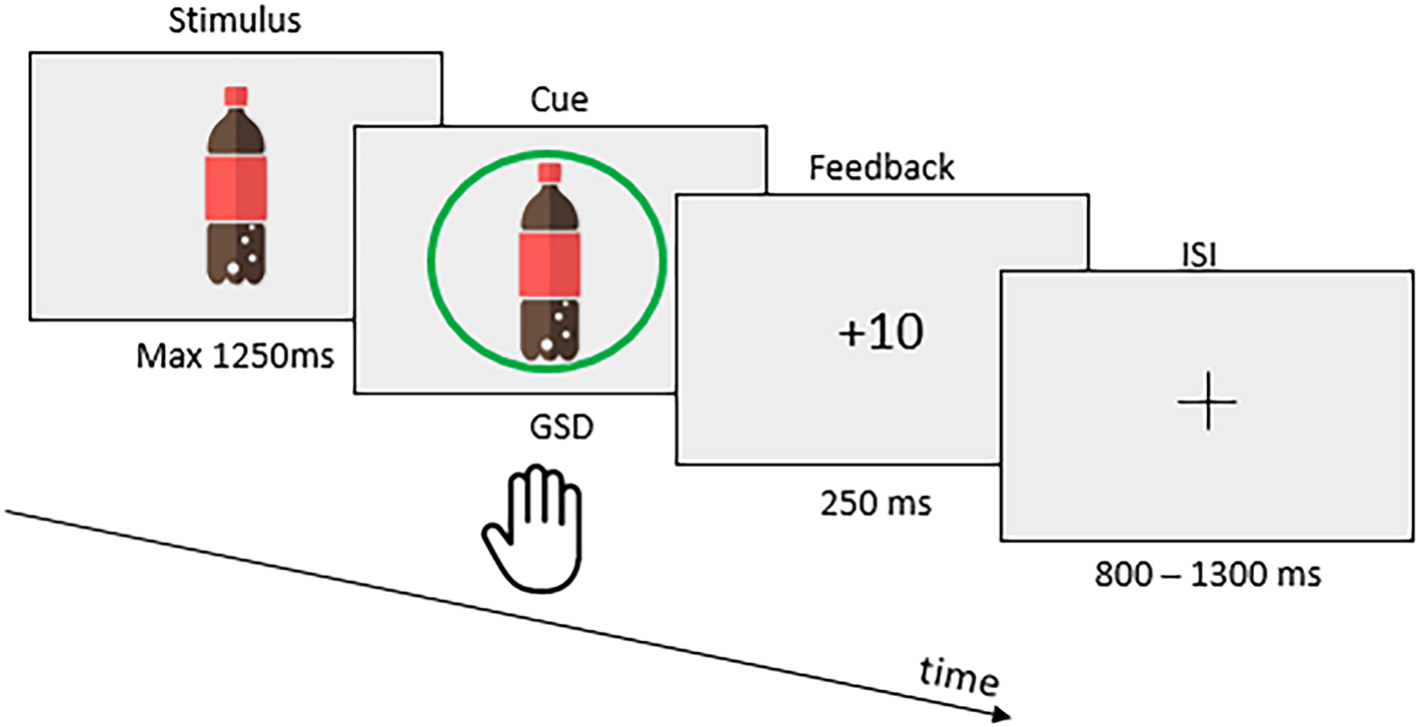
Schematic ABM task timeline, depicting the sequence of stimuli and response window.

In the ABM task, pictures appeared on the screen one after another at random locations on a grid. When a green cue was presented around the picture, accompanied by a bell sound, the participants had to click on the item before its offset occurred. If the participant responded between the cue onset and the item offset, a positive green feedback (the points obtained) was displayed with a rewarding sound. If they responded to a cued picture after the item’s offset, a negative orange (‘too late’) feedback was shown. If they did not respond to a cued picture or responded to a non-cued item, a negative red cross appeared as feedback. In the case of correct response withholding, a dark grey-green feedback was displayed with a neutral non-ascending sound, and a third of the hit point was awarded to avoid creating attentional bias during NoGo trials (Fig. 3).

#### Game design

##### Gamification

**Figure 4:**
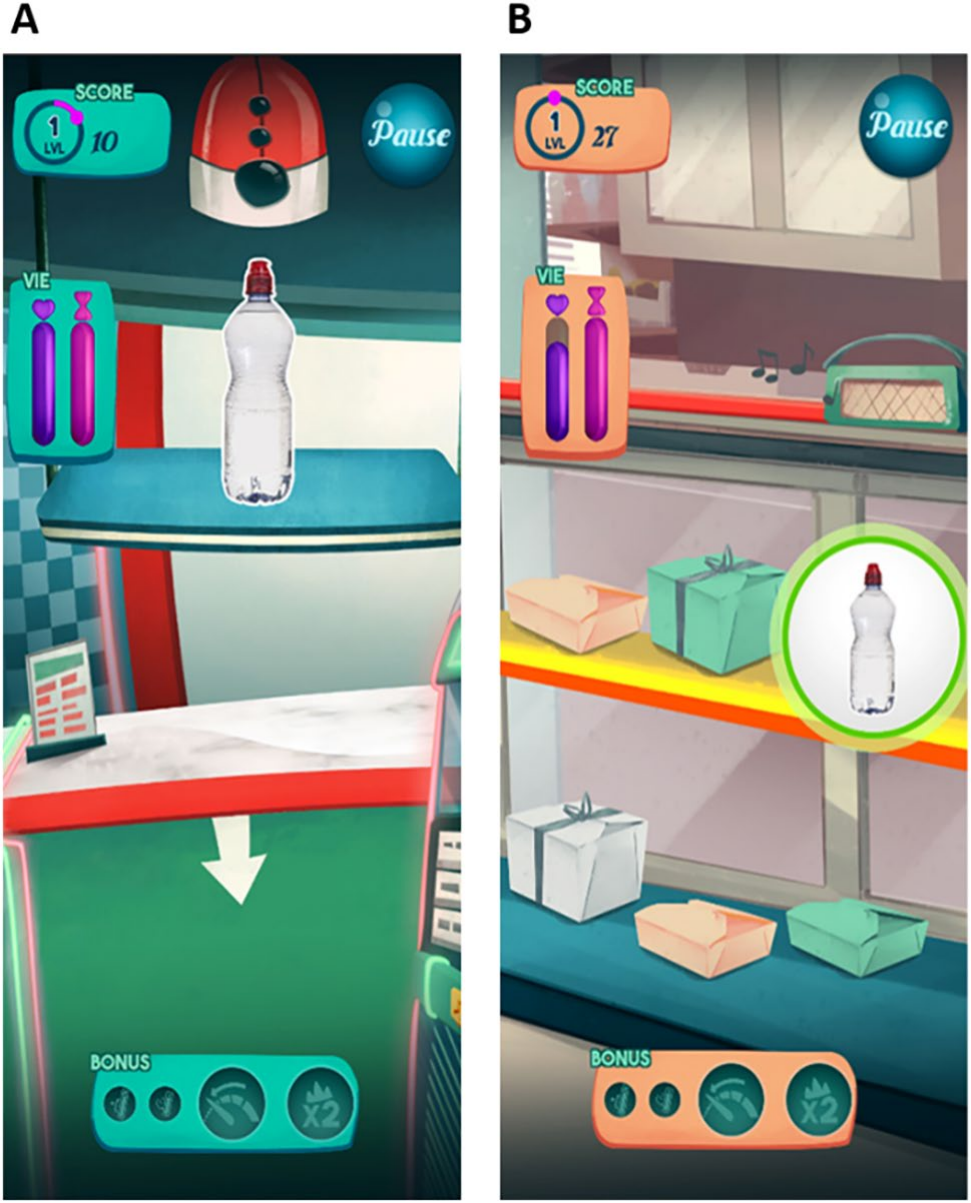
Screenshots of the in-game Go/NoGo (**A**) and ABM (**B**) tasks. The key gamification elements include visual and auditory arts, score and power-ups.

We maximised the participants’ motivation using state-of-the-art principles of game design. We included (1) challenging goals with progressive difficulty and a local and global ranking system to extract the score’s value; (2) different game modes to allow for gameplay diversity, further improved by an in-game currency mechanic to acquire power-ups; and (3) an artistic direction to create an enjoyable, rich experience for the players, in which all scenes have their own color and sound code to create a coherent universe (Fig. 4). Further, this artistic direction stresses reward-punishment mechanisms with specifically designed sounds.

##### Task mechanics

The aim of each task was to ‘survive’ for as long as possible while the difficulty continuously increased. After six correct responses during the GNG task, the difficulty was raised by decreasing the RTT. For the ABM, the difficulty was increased after three correct cued trials by moving the cue onset closer to the picture’s offset (Go Signal Delay; GSD), thus reducing the available response time window (Table 4). Importantly, these increases in time-pressure in turn raised the probability of committing a speed and/or commission error (FA). We designed the 18 difficulty levels to range from a very easy to a nearly impossible RTT close to the minimal physiological response time at the last difficulty level.

When a total of five speed or five commission errors were reached, the run was over. The current error count was displayed by a speed and accuracy gauge whereby a level was lost when an error was committed. This distinct gauge system was introduced to ensure that the participants maintain a stable speed-accuracy trade-off.

At the end of a run, the participants received feedback on their score, the ranking of their run compared to their previous run, in-game currencies, and the difficulty level they reached. In case of a best score, a rewarding animation was played. Further, a gauge displaying how many times they played (in minutes) was filled with the current length of the run. When this gauge was full, it meant the participant had completed the required 10 min of training time for this task. To complete the intervention, the participants needed to fill this gauge 20 times during each task for a total of 400 min.

Rewarded in-game currencies allowed the participants to renew their game experience by buying four kinds of ‘power-ups’ interacting with the number of permitted errors, difficulty, and score mechanisms. This currency was task-specific (i.e., to obtain a power-up at the ABM, currencies need to be obtained from the GNG task, and vice versa). More details about this procedure are available on the OSF webpage (10.17605/OSF.IO/5ESMP).

From the start menu screen, the participants could access the rank of their best scores compared to the other participants’ best scores in the shape of a table as an optional social motivation incentive.

#### Questionnaires

We assessed demographic information, as well as inclusion and exclusion criteria, with a custom-made, 9-item general health questionnaire. We determined high sugar-density drinking frequency in this questionnaire with the question adapted from Lawrence et al. (2015b)^41^: ‘At which frequency would you estimate your normal consumption of sugary drinks (soda, energy drinks, iced tea…)’ using an 8-point scale from 1 = 4 cans (≈1 bottle) or more per day, to 7 = 1–3 cans (less than a bottle) per month, and 8 = less than that or never. We included participants who stated drinking 2–4 cans a week or more.

Based on the following, we evaluated questions related to the interventions and condition expectations post-intervention:

- The Game Experience Questionnaire core module^35^ was translated into French as a measure of the motivational dimension of the intervention. This version, adapted to our study, contains 27 items on a 5-point Likert scale ranging from ‘not at all’ to ‘extremely’ and divided into 5 components: *competence*, *flow*, *tension/annoyance*, *challenge*, *negative affect,* and *positive affect*. We removed the *sensory and imaginative immersion* component from the published version as it does not apply to our study. By grouping the *competence*, *flow*, *challenge*, and *positive affect* component values with the opposite values of the *tension/annoyance* and *negative affect* components, we can obtain its global value.
- The custom-made, debriefing, post-intervention questionnaire contains five items about the participants’ feelings and understanding of the intervention’s purpose. We assessed the participants’ expectations of the intervention’s affects by asking the following question: ‘What do you think the game practice has improved or modified’? If their response included the notion of better soda drinking habits and/or modification of item ratings, the variable ‘expected valuation’ took a value of 1. If their response included the idea of reduced soda consumption, the variable ‘expected reduced consumption’ took a value of 1. Any other responses (not understanding the outcome, unhealthy item valuation increase, increase in soda consumption, etc.) resulted in a value of 0 for their respective variables.

### Sampling plan

For the ANOVAs (H1, H1a, H1b), we conducted power analyses based on a Monte Carlo approach^42,43^ to identify the minimum sample size required to reach 0.95 power with a 0.05 alpha in the interaction terms. This approach consisted of randomly drawing (here, 10′000 iterations) data points from normal distributions following estimated parameters (i.e., the between- and within-subject standard deviation [SD] and the smallest absolute effect size of interest), and then using these simulated datasets to compute the proportion of significant terms of interest at a given sample size, which corresponds to the power.

We used this approach for the H1 power analyses due to the complexity of the design, which precluded relying on the classical analytical approach as we did for H2 and H3.

#### H1) Reduction of unhealthy items and the increase in healthy items’ valuation

For H1, based on data from an ongoing Registered Report in our group using an intervention with similar tasks and for non-drink food items^34^ (see the ‘General Procedure’ section), we estimated the task within-subject sd to be 10/100 on the palatability VAS and the between subject SD to be 12/100, and we considered 5/100 points as the minimum change in interest for both healthy and unhealthy items. On this basis, 51 participants per group (102 in total) would be necessary to detect a 2 × 2 × 2 within-between ANOVA interaction 95% of the time.

For H1a and H1b, using the same parameters as H1), 106 participants per group (212 in total) would be necessary to detect a 2 × 2 within-between ANOVA interaction 95% of the time.

#### H2) Reduction in consumption over the days

For H2, based on a priori power analysis^44^ using G-Power^45^, the minimum sample size for our within-between ANOVA to reach a 0.95 power, with an alpha of 0.05, was 210 participants given a minimal Cohen’s f of interest of 0.25.

#### H3) The devaluation of unhealthy items’ ratings mediates the reduction in unhealthy item consumption

For H3, according to Vittinghoff, Sen, and McCulloch’s method^46^, the minimum sample size for our mediation model to reach a 0.95 power (alpha = 0.05) can be computed by estimating the smallest b2 of interest (0.1), the SD of the change in the palatability rating (10 points within-subject sd of palatability ratings), the standard deviation of the error (a conservative value of 3), and the correlation between the factor intervention and weight loss (0.3 average correlation size). On this basis, 129 participants in total would be needed using the R powerMediation package^47^.

A total of 210 participants is thus required to reach 0.95 power across all our contrasts. However, as the study was fully online, we could have reached a much larger sample size and potentially detected small, irrelevant effects. Hence, we decided to include additional criteria of minimum effect size for significant results to be interpreted. For H1, H1a, and H1b, we only interpreted a minimum mean difference of 5/100 between the control and experimental groups. For H2, the standardised effect size of interest (i.e., partial Cohen’s f) must reach at least 0.25 for the group-by-session interaction of our within-between ANOVA, corresponding to a medium effect^44^. For H3, both H1a and H2 should be above their respective minimum effect size of interest.

Should have we reached a higher sample size as the minimally required sample size, we would have stopped inclusion in the study at a total of n = 300.

The ‘Analysis plan’ below details the exclusion rules for data analysis and how they would affect our sampling plan.

### Analysis plan

We computed ANOVAs using the R base function, and computed mediation analysis using the mediation R package^48^.

Our alpha threshold is set to 0.05. We determined partial Cohen’s f as standardised effect sizes for ANOVAs using the R effect size package^49^.

For each model, we tested the homoscedasticity assumption using the R car package^50^. If the assumption was violated (Levene test with p < 0.05), we employed a non-parametric approach (Brunner and Langer’s non-parametric, mixed-effects models^51,52^) for the corresponding analysis.

Bayes Factors were computed using the R BayesFactor package^53^, and reported for every ANOVA. The models, priors, and methods of computation are provided in Rouder et al. (2012)^54^.

#### Data exclusion rules

We excluded participants from all analyses if they completed less than five sessions of training.

##### Palatability ratings

We excluded all palatability ratings from a given participant (from the analyses) if the participant did not complete the pre- or post-intervention palatability rating questionnaires.

To ensure a thorough filling of the analogue scales, we excluded items with an RT shorter than 300 ms. We only considered trained items (those above the median before the intervention) in the analysis. For each participant, we calculated the mean ratings after trimming the 20% highest and the 20% lowest rated items at pre-intervention from the healthy and unhealthy item distribution, to only target the items with room to change, thus preventing ceiling and floor effects.

We defined outlier participants as those outside the 2.5*MAD range around the median (median average deviation; moderately conservative criterion^55^) for each intervention group at both sessions, and removed them from the related descriptive and inferential analyses.

##### Consumption

We followed the same rationale to detect outlier participants than for the palatability ratings (i.e., 2.5*MAD around the median at pre- and post-session). We removed the outlier participants from the related descriptive and inferential analyses. Please note that the consumption analysis was first designed to be a linear regression, but was changed to a 2 × 2 ANOVA after Stage 1 review. We forgot in the stage 1 manuscript to modify the outlier detection rationale to reflect this change.

#### Statistical contrasts and predictions

##### H1) Modification in palatability rating

We assessed the modification in the participants’ palatability ratings by the triple interaction term of the intervention (experimental vs. control) × session (pre-, post-intervention) × drink type (healthy; unhealthy) mixed ANOVA.

As for planned contrasts, we then split this interaction into two intervention × session mixed ANOVAs, with the unhealthy palatability rating (H1a) and the healthy palatability rating (H1b) as dependent variables.

##### H2) Reduction in consumption

We assessed the change in high sugar-density drink consumption by the interaction term of the intervention (experimental vs. control) x session (pre-, post-intervention) mixed ANOVA. We used the first and last consumption data as pre- and post-measures, respectively. We predicted a larger decrease in consumption in the experimental group than in the control group.

##### H3) Changes in palatability ratings mediate changes in consumption

If both ANOVAs on the palatability ratings and consumption reached significance, were driven by a greater effect for the experimental versus the control group, and were above our minimum effect size of interest, we would have performed a causal mediation analysis with 10,000 Monte Carlo draws for quasi-Bayesian approximations, with the intervention factor as the independent variable, the change in consumption as the dependent variable, and the change in unhealthy palatability ratings as the mediator. If the average causal mediation effect (i.e., the indirect effect) is significant, then we would have interpreted changes in unhealthy palatability ratings as mediating the effect of the intervention on reduced consumption.

#### Positive controls and quality checks

##### Between-group baselines

To verify that the intervention condition assignment resulted in sufficiently balanced baselines*,* we checked that the experimental and control group both showed a pre-intervention difference of Cohen’s d below 0.4 for the weight, age, the trained unhealthy items’ rating and the trained healthy items’ rating, in addition to an odds of gender ratio (M/F in the experimental group divided by M/F in the control group) below 1.4 or above 0.7 (please note that the “below” and “above” signs were reversed in Stage 1 by mistake).

Since we implemented a selection of the target items based on the participants’ individual ratings and since the assignment was randomised, we did not test with inferential statistics whether these values were equivalent between the two groups. As such, we did not conduct a power analysis for these effects.

##### Primary outcome statistical assumptions

Any biasing of the primary statistical outcomes by ceiling or floor effect was controlled on the palatability ratings by using trimmed means (see the ‘Data exclusion’ section) and by employing an additional non-parametric statistical approach (Brunner and Langer’s non-parametric mixed-effects models^51,52^) in the case that the homoscedasticity assumption is violated.

##### Training duration

To ensure that the effect of the intervention between control and experimental group were not biased by how long participants trained, we ensured that there was only a small difference (Cohen’s d < 0.4) between the average total playtimes between each group. In the case of a large difference (d ≥ 0.4), we excluded the participants with the most extreme values until the maximum threshold is reached. This positive control was added after we modified the training length recommendation; this modification received editorial approval before the beginning of data collection.

##### Game experience

To avoid between-group imbalance in motivation, we only tolerated a small difference (Cohen’s d < 0.4) between the global index of the game experience questionnaire of the experimental and control groups. In the case of a larger difference (d ≥ 0.4), we excluded the participants with the most extreme values until the maximum threshold is reached.

##### Expectations

We used count data’s 2-by-2 contingency matrices of the ‘expected valuation’ and ‘expected reduced consumption’ binary variables (see the ‘Questionnaires’ section) to measure the between-group balance in expectations (in the Stage 1 manuscript, the variable ‘expected reduced consumption’ appearing here was mistakenly referred to as ‘expected weight loss’). If the phis of the contingency matrices were below or equal to 0.2 (a small effect size), we considered the value low enough so that both groups can be considered balanced. Otherwise, we excluded and replaced random participants from the unbalanced cells until reaching this threshold.

## Results

### Participants

**Figure 5:**
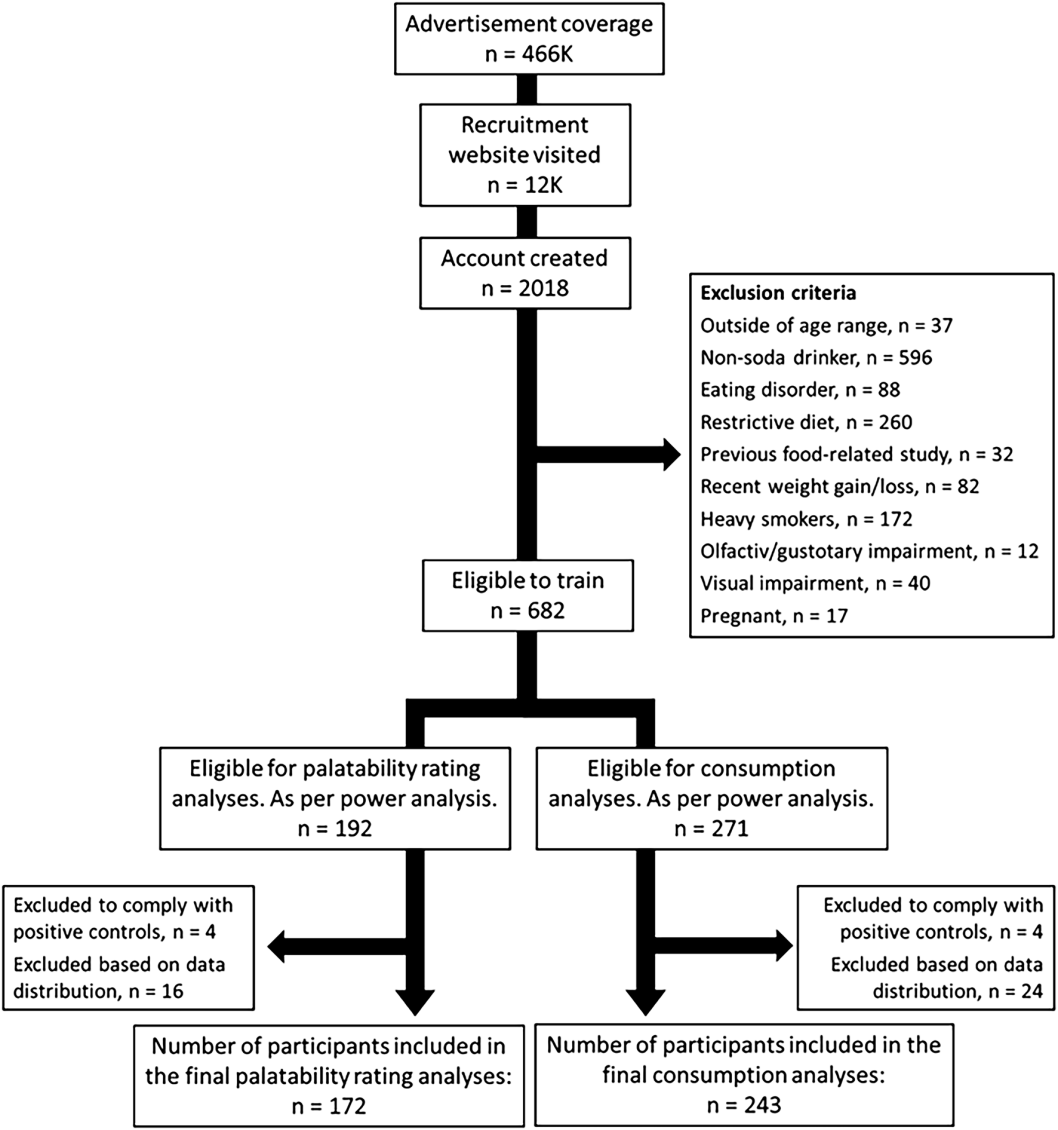
Data exclusion flowchart, including the number of participants excluded at each step of the data processing. The participants included in the palatability rating analyses were also included in the consumption analyses.

A total of 2018 participants consented to participate to the study online. Of them, 682 were eligible after the exclusion criteria. 271 participants completed the minimum five days of training required to be to be included in the consumption analyses, and of them, 192 completed the post-intervention palatability questionnaire required for the palatability analyses (Fig. 5; see Table 5 for demographic data).

**Table 5:** Demographic data.

Mean ± SD	Control (n = 142)	Experimental (n = 129)
Age	30.1 ± 7.1	29.3 ± 6.6
Gender ratio (M/F)	0.42 (42 M/100 F)	0.36 (34 M/95 F)
Weight (kg)	75.3 ± 18.6	73.7 ± 17.4
Trained days (of participants who completed the training)*	12.7 ± 5.8 (n = 93)	13.8 ± 5.8 (n = 78)

*Due to an unidentified bug, 21 participants (13 control, 8 experimental) did not have their amount of played time sent to our server.

As our Monte Carlo power analyses provided the necessary sample size before quality exclusions (i.e., distribution outliers and positive controls), the sample sizes to take into consideration are 192 for H1 and 271 for H2.

### Positive controls

All but the positive controls on the intervention’s expectations were met with the initial sample (phis above 0.2). After randomly excluding the outlier participants (three participants of the control intervention and one of the experimental interventions; see “Positive controls and quality checks” section), the positive controls criteria were all met. Detailed results of the positive controls and Game Experience Questionnaire can be found in the online supplementary materials (https://osf.io/v78ke?view_only=b671191261424e80bd780fa0b5909ab3).

### H1) Palatability ratings

**Figure 6:**
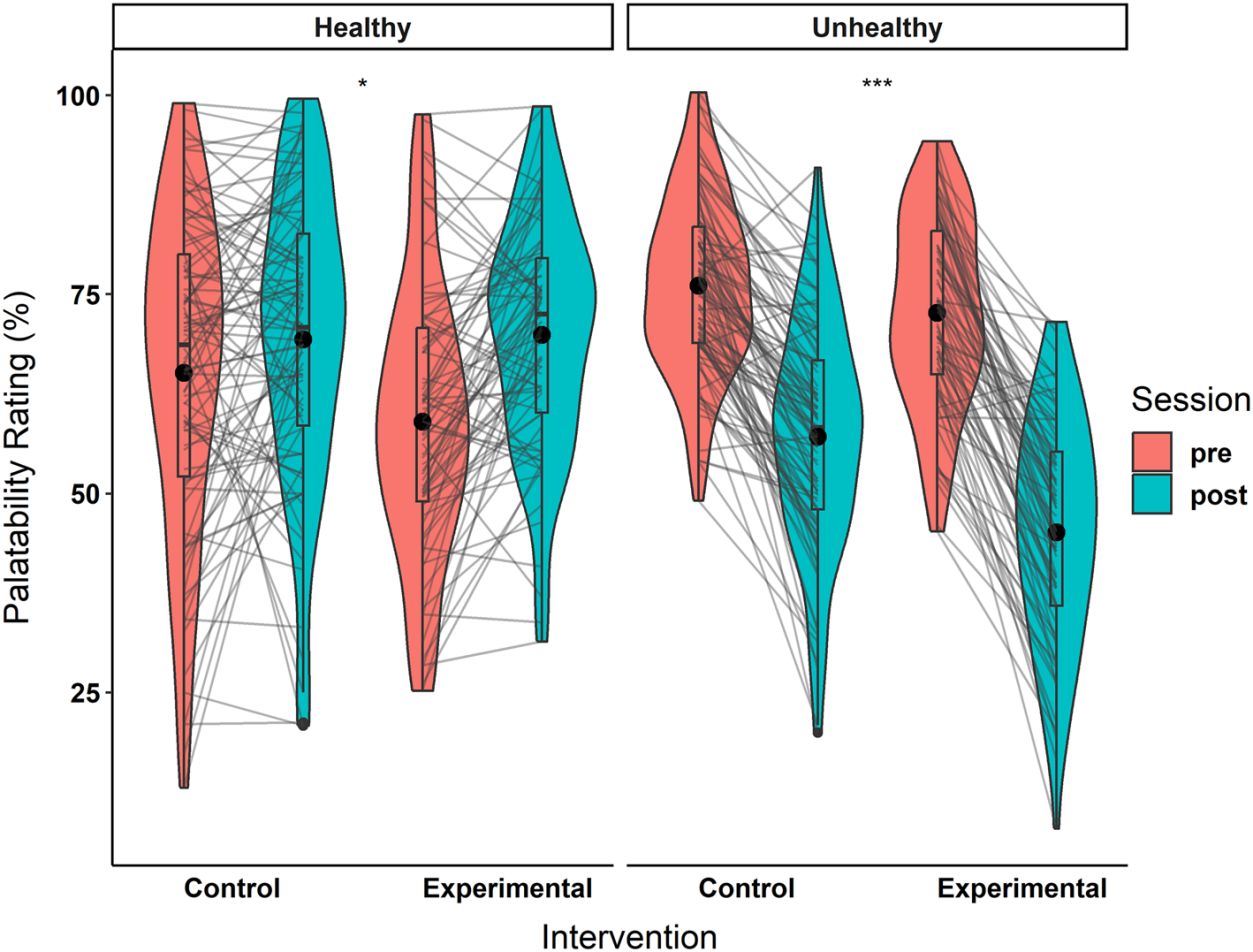
Palatability ratings at pre- and post-intervention. Trimmed means of palatability ratings are represented for the control and experimental interventions. Individual data points, means (bold circle), distributions’ density (violin), medians, first and third quartiles (horizontal bars), and the 1.5 inter-quartiles range (whiskers) are represented. *p < 0.05, **p < 0.01, ***p < 0.001.

Results of the palatability ratings are reported in Table 6 and Fig. 6.

**Table 6:** Palatability ratings results.

Mean ± SD	Control Intervention (n = 94)		Exp Intervention (n = 78)		Intervention x Session	Intervention x Session x Drink Type
	Pre	Post	Pre	Post		
Healthy item ratings (%)	65.1 ± 20.2	69.3 ± 18.7	59 ± 17.2	69.9 ± 14.7	f = 0.18 p = 0.02	f = 0.32 p < 0.001
Pre-Post delta [0.95CI]	4.2 [0.1; 8.3]*		11 [7.1; 14.7]***			
Unhealthy item ratings (%)	76.1 ± 11	57.1 ± 14.3	72.7 ± 12	45.1 ± 14.2	f = 0.31 p < 0.001	
Pre-Post delta [0.95CI]	− 19 [− 21.8; − 16.2]***		− 27.6 [− 30.1; − 24.3]***

For the triple interaction (H1) Intervention x Session x Drink Type, the homoscedasticity assumption was not respected (Levene’s Test: F(7, 680) = 5.8, p < 0.001). Hence, a non-parametrical ANOVA was computed. It revealed a significant triple interaction, driven by a different training effect between healthy and unhealthy items’ palatability ratings (Statistic(1, 169.89) = 16.2, p < 0.001).

The triple interaction was split into two follow-up Intervention x Session interactions for the unhealthy (H1a) and healthy (H1b) drink items separately. Both of these double interactions had their homoscedasticity assumption respected (H1a: F(3, 340) = 2.3, p = 0.08; H1b: F(3, 340) = 2.4, p = 0.07). Confirming our hypotheses for both the unhealthy (H1a) and healthy (H1b) items, the interactions reach significance and with above-threshold effect sizes (i.e., differences of pre-post deltas > 5%). The interaction was driven by a larger reduction of palatability ratings in the experimental than control intervention for the unhealthy items (delta of pre-post deltas = 8.6, F(1, 170) = 16, p < 0.001), and by a larger increase in the experimental than control intervention for the healthy drinks (delta of pre-post deltas = 6.8, F(1, 170) = 5.4, p = 0.02).

### H2) Sugary drinks consumption

**Figure 7:**
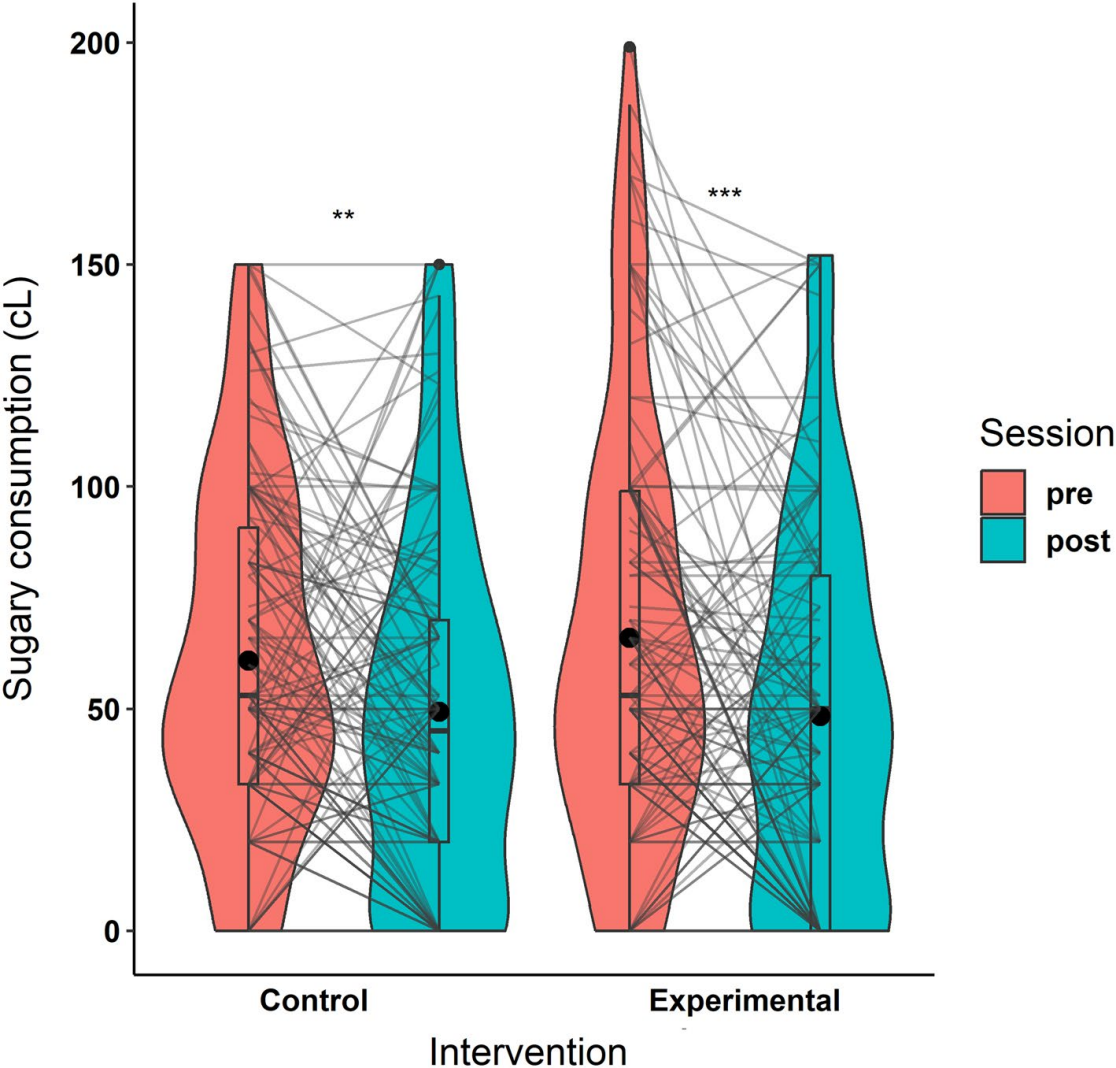
Sugary drinks consumption in cL at pre- and post-intervention for the control and experimental interventions. Individual data points, means (bold circle), distributions’ density (violin), medians, first and third quartiles (horizontal bars), and the 1.5 inter-quartiles range (whiskers) are represented. *p < 0.05, **p < 0.01, ***p < 0.001.

Results of the sugary drink consumption are reported in Table 7 and Fig. 7.

**Table 7:** Self-reported sugary drinks consumption results.

Mean ± SD	Control intervention (n = 124)		Exp intervention (n = 119)		Session	Intervention × session
	Pre	Post	Pre	Post		
Consumption (cL)	60.9 ± 39.2	49.4 ± 41.2	66 ± 47.9	48.3 ± 42.1	f = 0.3 p < 0.001	f = 0.06 p = 0.32
Pre-Post delta [0.95 CI]	− 11.5 [− 19.5; − 3.5]**		− 17.6 [− 27.9; − 8.4]***

For the Intervention x Session interaction on self-reported sugary drinks consumption, the homoscedasticity assumption was respected (Levene’s Test: F(3, 482) = 1, p = 0.42). The double interaction did not reach significance and was below our minimal effect size of interest (i.e., partial f < 0.25), with its Bayes Factor providing support for an absence of interaction (partial f = 0.06, F(1, 241) = 0.99, p = 0.32, BF_01_ = 4.7). Contrary to our hypotheses, the reduced consumption was not larger in the experimental than control intervention. However, we observed a significant effect of Session (partial f = 0.3, F(1, 241) = 22.2, p < 0.001), driven by a reduction of consumption in both the experimental (pre-post delta = − 17.6) and control interventions (pre-post delta = − 11.5).

### H3) Valuation to consumption mediation analyses

Because the hypothesis on sugary drinks’ consumption was not validated, the mediation analysis between palatability ratings and consumption (H3) was not performed (cf the “Materials and methods” section).

## Discussion

In this registered report, we identified the effect of a fully online, app-based gamified food executive control training (ECT) combining a Go/NoGo (GNG) and an attentional bias modification (ABM) task, practiced for 20 min a day for five to twenty days. We used a double-blinded randomized controlled trial in which participants were either assigned to a control or an experimental group differing only at the level of the association between unhealthy sugary drink visual cues and the inhibition of motor response (100% association in the experimental group; 50% association in the control group). Confirming our hypotheses, and in line with previous reports from our^34^ and other groups^9–12^, we found a larger decrease in the explicit liking of the target unhealthy items in the experimental than in the control group with training (− 28% vs. − 19%). For the healthy (water) trained items, we observed a larger increase in the experimental than in the control group (11% vs. 4.2%), which replicates the typical effect of attentional bias modification interventions^18,20–22^. Contrary to our hypotheses, we did not find any evidence for a larger reduction in self-reported measures of drink consumption in the experimental than in the control group (BF_01_ = 4.7), while still observing a global reduction in consumption in both groups (control: − 11.5 cL [− 19%]; exp: − 17.6 cL [− 27%] per day).

Because baseline items valuation and the expectation on the effect of the intervention were similar between the control and experimental groups (phis < 0.2), we can conclude that the difference between the two groups only follows from the variation in the probability of mapping between the sugary drink cues and response inhibition.

### Motoric inhibition and execution modify items’ valuation

A decrease in the valuation of items associated with response inhibition is the most robust effect found in food-ECTs, even after a single twenty-minute session^27,28,30^. The replication of this effect in the present study first indicates that the innovative aspects of our online intervention, notably its high-level gamification, did not interfere with the core ‘action-to-valuation’ mechanisms of action at play in food-ECT. We thereby confirm that the effect of response inhibition on items valuation remains robust and large across various experimental settings.

The increase in the healthy items’ valuation (11% in the experimental vs. 4% in the control intervention) was smaller than the decrease in the unhealthy items’ valuation. We interpret this pattern as following from a ceiling effect instead of a smaller impact of the training on the items associated with response execution than inhibition. Indeed, the healthy (Go) items were highly liked by the participants from the start (baseline mean = 0.62, sd = 0.19), and thus had little room to increase, in contrast to the unhealthy items’ ratings which were also highly liked and could thus decrease more (baseline mean = 0.75, sd = 0.11). Of note, this pattern replicates the increase in valuation seen in the ABM literature^18,22^, and support that even when combined, GNG and ABM training deploy their own effects without interference^25,34^. Further studies are however required to determine whether and how the valuation of disliked item may increase, and if training combination improve ECT efficacy across various types of items (meat, cigarettes, alcohol, etc.).

### Motoric inhibition reduces the consumption of sugary drinks

Regarding our result for an unspecific effect of the intervention on sugary drink consumption, previous data suggest that self-reported measures of food intake can fail to detect real-world effects since participants’ ideas of what they consume can be biased^56^. We tried to mitigate this effect by focusing on the measure of sugary drinks’ consumption, whose packaging is highly standardized (e.g. 33 cl for a can, 50cL for a small bottle, etc.) and are rarely shared with peers. This choice of design allowed us to detect pre- to post- training reductions in both groups, but no larger reduction in the experimental than control. This indicates that the effect of a coherent (100%) mapping of drink cues with motor inhibition did not lead to effects larger than those of expectations and of the 50% mapping of the control intervention. However, we cannot exclude memory biases in consumption recollection^57^, especially in individuals with higher Body Mass Indexes.

To assess if the decrease in sugary drinks’ valuation translated into a reduction in their self-reported consumption, we conducted an exploratory analysis of the correlation between both variables (see Supplementary Material). We found no association between the reported decrease in consumption and devaluation (r = 0.1, Supp. Fig. 2), expressing that either our measure of consumption did not detect a real-world effect, or that the explicit liking of an item is independent of its consumption. Such cases are observed in various types of addiction, where item consumption and wanting (craving) can be dissociated from their liking^58^.

### Training-induced devaluation effect lasts up to 1 month

Our intervention was fully online with limited control over participants’ behavior. While the training app guided the participants through the steps of the study, the pace of the training was only encouraged and never enforced. As a result, 60 (35%) participants filled the post-intervention questionnaires up to 41 days after their last day of training (median [Q1–Q3] = 9 [4–16.5] days). We exploited this variability of the delay between the post-intervention questionnaires and the last day of training to assess the persistence of the training’s effect. A linear fit shows no evidence of an effect of the delay on the decrease in unhealthy valuation (b_1_ = 0.13%; Supp. Fig. 3), indicating that the effect of the intervention lasted at least 40 days. This finding is in line with previous reports for food ECTs showing effects months after the end of training^59^. Moreover, there was no correlation between the amount of training and the devaluation effect (r = − 0.14, Supp. Fig. 4). We interpret these exploratory results as supporting that increasing the length of training beyond 5 days does not increase the effect size of the intervention. It could however impact how much its effects persist.

### The challenge of large-scale online executive control training

While online training apps clearly help to conduct intervention for food intake behavior modification in large sample and with large amount of training, they do not come without important challenges. We notably experienced difficulties with OS updates blocking data communication with our servers, app stores changing their security requirements, and participants not following the intervention procedure if not completely enforced by the app itself. Anticipating these issues may help to complete future corresponding studies.

## Conclusions

The present registered report reveals that five to twenty days of combined practice of a GNG and ABM tasks robustly reduces the perceived value of the trained unhealthy sugary drink cues and their reported consumption. The decrease in consumption did not follow a dose-dependent relationship with the consistency of the mapping between the targeted items and motor inhibition. Our strict positive controls of baseline states and expectations, together with our use of a mechanistic control group, ensure that the effect observed on item valuation only follows from an “action-to-valuation” mechanism of action. Exploratory analyses suggest that the length of the intervention did not increase its effect size, but its persistence in time. In addition to confirming this latter observation, future studies may examine the effect of ECTs on implicit measures of liking/wanting and on various consumption outcomes, to provide conclusive information on whether such an approach indeed impact real-world behavior beyond perceived food item values.

## Supplementary Information

Supplementary Information.

## Data Availability

Raw data and materials are accessible on our OSF page (10.17605/OSF.IO/5ESMP).

## Code availability

All scripts are accessible on our OSF page (10.17605/OSF.IO/5ESMP).
