# Supplementary material for: Reduction in sugar drink valuation and consumption with gamified executive control training
Source: Sci Rep. 2023 Jun 30;13:10659. doi: 10.1038/s41598-023-36859-x (PMC10313656; doi:10.1038/s41598-023-36859-x)
Supplement: Supplementary file 1 — Supplementary Information. [file 41598_2023_36859_MOESM1_ESM.docx]

# Supplementary material

## Game Experience Questionnaire

There are no differences in scores for the Global Experience Questionnaire between the control and experimental interventions (d = .01), as detailed in the supplementary table and figure 1.

Overall, the game received a global score at the middle of the modalities’ average, showing a decent appreciation of the game by the participant. All components were above their modalities’ averages, at the exception of the flow and challenge components.

***Supplementary Table 1****. Game Experience Questionnaire results*

| **Mean ±SD** | **Global Score** | **Competence** | **Flow** | **Tension (reversed score)** | **Challenge** | **Negative affects (reversed score)** | **Positive affects** |
| --- | --- | --- | --- | --- | --- | --- | --- |
| **Control (n=98)** | 53.7 ±10.7 | 10.3 ±3.8 | 7.4 ±4.4 | 10.4 ±4.3 | 7.3 ±3.9 | 8.5 ±3.7 | 9.6 ±4.3 |
| **Experimental (n=78)** | 53.7 ±10.2 | 10.8 ±4 | 7 ±4.1 | 10.2 ±4.1 | 6.9 ±3.6 | 9 ±3.5 | 9.8 ±4.1 |


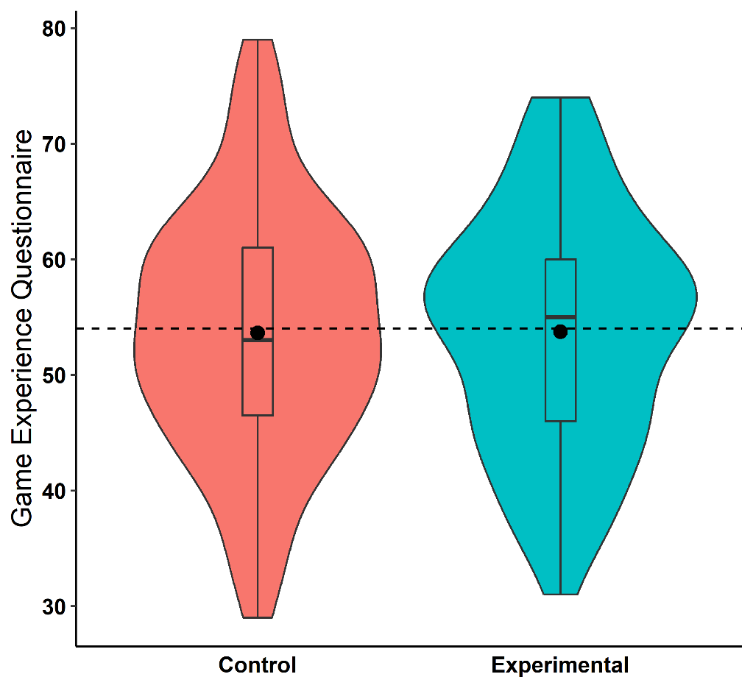


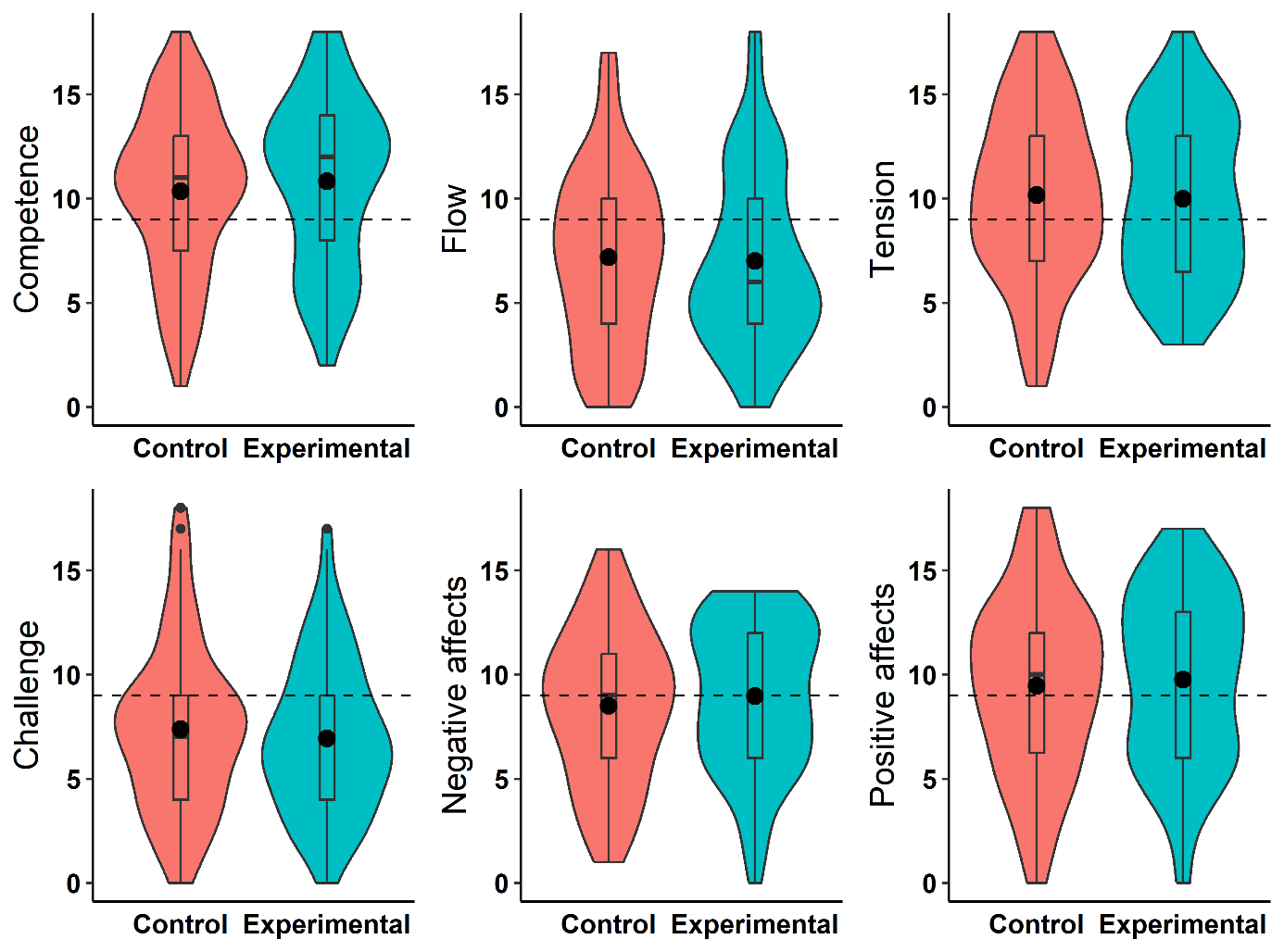


***Supplementary Figure 1****. Game Experience Questionnaire. Both Control and Experimental groups are represented for all scores. Means (bold circle), distributions’ density (violin), medians, first and third quartiles (horizontal bars), and the 1.5 inter-quartiles range (whiskers) are represented. The modalities’ average is represented by dashed horizontal lines. Both tension and negative affects components are reversed, with a higher score representing a better experience.*

## Positive controls

All but the positive controls on the intervention expectations were respected (phis above 0.2). After randomly removing participants in the outlier cells (three participants of the control intervention and one of the experimental intervention), the positive controls are all respected.

Before quality exclusion:

- Weight, d = .02
- Age, d = -.05
- Gender, M/F odd ratio = 1.1
- Baseline unhealthy rating, d = -.1
- Baseline healthy rating, d = -.3
- Average play time, d = .33
- GEQ, d = .03
- Expected reduced valuation, phi = .21
- Expected reduced consumption, phi = .21

|  | **No expectation of an effect** | **Expectation of an effect** |
| --- | --- | --- |
| **Control** | 52 | 52 |
| **Experimental** | 24 | 57 |

***Supplementary Table 2a.*** *Contingency matrix on the expectation of healthier items’ valuation*

|  | **No expectation of an effect** | **Expectation of an effect** |
| --- | --- | --- |
| **Control** | 33 | 71 |
| **Experimental** | 11 | 70 |

***Supplementary Table 2b.*** *Contingency matrix on the expectation of reduce sugary drinks’ consumption*

The outlier cells are “Control + No Expectation” and “Experimental + Expectation”. By randomly removing three and one participants in these cells respectively, the positive controls were all respected as described below.

After quality exclusion:

- Weight, d = .03
- Age, d = -.03
- Gender, M/F odd ratio = 1.1
- Baseline unhealthy rating, d = -.16
- Baseline healthy rating, d = -.33
- Average play time, d = .27
- GEQ, d = .01
- Expected reduced valuation, phi = .19
- Expected reduced consumption, phi = .19

## Exploratory analysis

## *Link between the effect of unhealthy items’ devaluation and consumption*

No linear link is observed between the pre-post delta of unhealthy items’ ratings and the pre-post delta of the self-reported consumption (.r [95% CI] = .1 [-.06 ; .25], t(155) = 1.25, p =.21)


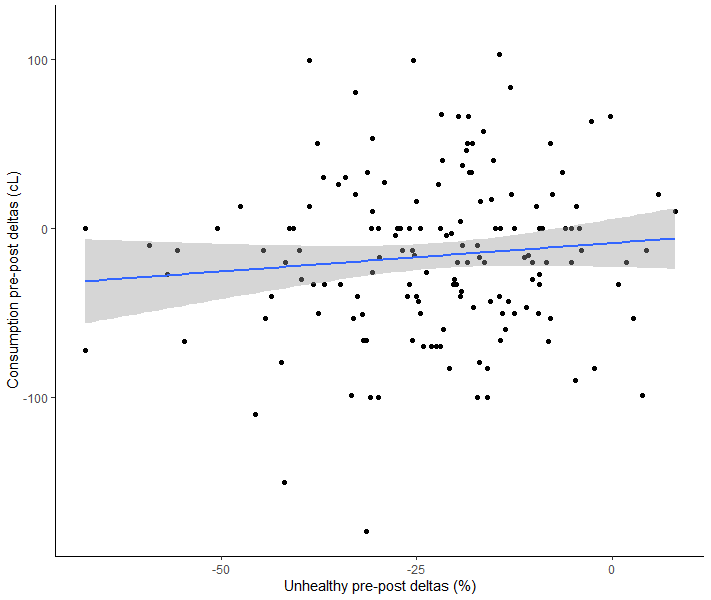


***Supplementary Figure 2*** *Pre-post deltas of unhealthy items’ ratings in % (x-axis) and reported-consumption in cL (y-axis) are plotted for each participant (dots). The linear model’s projection (blue line) and its standard error (grey area) are represented.*

## *Effect of time on the intervention*

By utilizing the time some participants took to complete the palatability questionnaire after their last training, no link was found between the time after training and the main effect of intervention (i.e., the unhealthy items’ devaluation; .r [95% CI] = .06 [-.09 ; .22], t(151) = .75, p =.5)


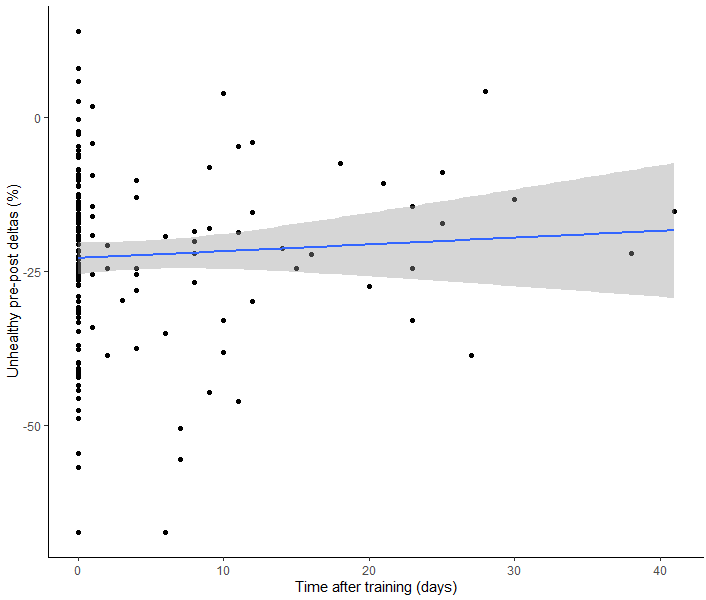


***Supplementary Figure 3*** *The time gap after the last day of training in days (x-axis) and the pre-post deltas of unhealthy items’ ratings in % (y-axis) are plotted for each participant (dots). The linear model’s projection (blue line) and its standard error (grey area) are represented.*

This absence of correlation between the time after training and the effect on unhealthy palatability ratings goes into the direction of a long-lasting effect of training.

Furthermore, no link was found between time spent playing and the unhealthy items’ devaluation (.r [95% CI] = -.13 [-.28 ; .03], t(152) = -2.8, p =.1), showing that the length of the intervention does not increase its effect size.


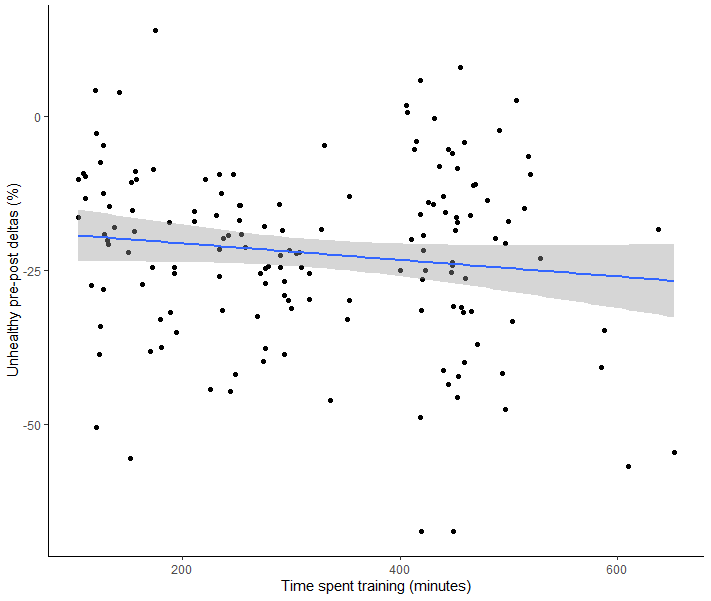


***Supplementary Figure 4*** *The time spent training in minutes (x-axis) and the pre-post deltas of unhealthy items’ ratings in % (y-axis) are plotted for each participant (dots). The linear model’s projection (blue line) and its standard error (grey area) are represented.*
